# Enhancement of chondrogenic differentiation supplemented by a novel small compound for chondrocyte-based tissue engineering

**DOI:** 10.1186/s40634-020-00228-8

**Published:** 2020-03-07

**Authors:** Shuichi Hamamoto, Ryota Chijimatsu, Kazunori Shimomura, Masato Kobayashi, George Jacob, Fumiko Yano, Taku Saito, Ung-il Chung, Sakae Tanaka, Norimasa Nakamura

**Affiliations:** 1 Orthopaedic Surgery, Osaka University Graduate School of Medicine, Suita, Japan; 2 Bone and Cartilage Regenerative Medicine, The University of Tokyo, Tokyo, Japan; 3 Center for Disease Biology and Integrative Medicine, The University of Tokyo, Tokyo, Japan; 4 Sensory and Motor System Medicine, The University of Tokyo, Tokyo, Japan; 5 Global Center of Medical Engineering and Informatics, Osaka University, Suita, Japan; 6 Institute for Medical Science in Sports, Osaka Health Science University, Osaka, Japan

**Keywords:** Chondrocytes, TD198946, Scaffold, Engineered cartilage

## Abstract

**Purpose:**

Chondrocyte -based tissue engineering has been a promising option for the treatment of cartilage lesions. In previous literature, TD198946 has been shown to promote chondrogenic differentiation which could prove useful in cartilage regeneration therapies. Our study aimed to investigate the effects of TD198946 in generating engineered cartilage using dedifferentiated chondrocyte-seeded collagen scaffolds treated with TD198946.

**Methods:**

Articular chondrocytes were isolated from mini pig knees and expanded in 2-dimensional cell culture and subsequently used in the experiments. 3-D pellets were then cultured for two weeks. Cells were also cultured in a type I collagen scaffolds for four weeks. Specimens were cultured with TD198946, BMP-2, or both in combination. Outcomes were determined by gene expression levels of RUNX1, SOX9, ACAN, COL1A1, COL2A1 and COL10A1, the glycosaminoglycan content, and characteristics of histology and immunohistochemistry. Furthermore, the maturity of the engineered cartilage cultured for two weeks was evaluated through subcutaneous implantation in nude mice for four weeks.

**Results:**

Addition of TD198946 demonstrated the upregulation of gene expression level except for ACAN, type II collagen and glycosaminoglycan synthesis in both pellet and 3D scaffold cultures. TD198946 and BMP-2 combination cultures showed higher chondrogenic differentiation than TD198946 or BMP-2 alone. The engineered cartilage maintained its extracellular matrices for four weeks post implantation. In contrast, engineered cartilage treated with either TD198946 or BMP-2 alone was mostly absorbed.

**Conclusions:**

Our results indicate that TD198946 could improve quality of engineered cartilage by redifferentiation of dedifferentiated chondrocytes pre-implantation and promoting collagen and glycosaminoglycan synthesis.

## Background

It is well known that articular cartilage injuries do not heal spontaneously due to cartilage tissue having a limited self-regeneration capacity. This has meant treatment strategies have remained challenging and now therapies have focused on tissue engineering for effective chondral injury treatment. Several biological strategies have been investigated and each are in different phases of development and vary in levels of evidence [10]. One accepted biological regenerative strategy is autologous chondrocyte implantation and this is associated with some amount of joint morbidity due to the requirement of cartilage biopsy from a normal region of the joint [14]. In order to reduce the harvest quantity primary culture of harvested chondrocytes and repeated passages must be performed to attain an adequate cell number. Repeat passaging leads to dedifferentiation of chondrocytes which has proven to result in fibrocartilaginous tissue [5, 9, 20]. A newer strategy involves preculturing the chondrocyte cells on a membrane which can help preserve their phenotype prior to implantation resulting in better regenerative tissue [4, 8].

Another method to improve the quality and efficiency of chondrocyte repair would be with use of a potentiating molecule or drug [3, 15]. We identified a small compound, TD198946 and demonstrated its positive effect in chondrogenic differentiation of C3H10T1/2 cells, ATDC5 cells, and primary mouse chondrocytes [19]. TD198946 has recently come to attention as a potential candidate as a disease-modifying osteoarthritis drug. It has been shown to prevent osteoarthritis progression in a mouse model and increase extracellular matrix (ECM) deposition through upregulation of Runx1 and Sox9 in two-dimensional cell cultures [19].

Our study aimed to investigate the effects of TD198946 in generating engineered cartilage using dedifferentiated chondrocyte-seeded collagen scaffolds treated with TD198946. We hypothesized that the addition of TD198946 to a three-dimensional culture or engineered cartilage would improve chondrocyte differentiation and therefore the quality of cartilage regenerate. This would provide some early evidence as to whether TD198946 have an important role to play in future tissue engineering techniques.

## Methods

Implantation protocol for nude mice was approved by the committee on the Ethics of Animal Experiments in Osaka University(Authorization number; 27–065-010).

### Harvest and isolation of articular chondrocyte

Cell isolation protocol for mini pig articular chondrocytes was followed as described below. Total number of mini pigs used for the study was 6. Porcine cartilage was obtained aseptically from the knee joints of skeletally mature male mini pigs (24 months of age) within 24 h of death. Knee joint articular cartilage was minced into small pieces and pre-treated with 0.1% pronase (Roche Diagnostics GmbH, Germany) in Dulbecco’s Modified Eagle Medium (DMEM) (Gibco BRL, Grand Island, NY), containing 10% fetal bovine serum (FBS) (Sigma-Aldrich, St Louis, MO, USA) for 20 min incubating at 37 °C. After centrifugation, participated tissue fragments were resuspended in DMEM (Gibco) containing 10% FBS (Sigma-Aldrich, St Louis, MO, USA) supplemented with 400 unit/mL collagenase type II (Worthington, Lakewood, NJ, USA) and incubated at 37 °C. After 12 h, dissociated cells were washed three times and cultured in tissue culture dish (Corning, Corning, NY, USA) with high glucose-DMEM containing 10% FBS and 1% antibiotic-antimitotic (Sigma-Aldrich) at 37 °C with humidified 5% CO_2_. Cells were passaged with 0.25% trypsin/EDTA at 80% confluency (about once per week) and replated at a density of 10,000 cells/cm^2^. Media changes were performed twice a week [13, 16]. Expanded articular chondrocytes were used for experiments at passage 3 (P3; at day 28). Articular chondrocytes at passage 0 (P0; at day 7) isolated from mini pig #4 were used for evaluation of gene expression level.

### 3-D pellet culture

3-D pellets were cultured with chondrogenic medium for two weeks in the presence of TD198946 1 nM to 100 nM for histological evaluation, and 1 nM to 1000 nM for qualification of glycosaminoglycan (GAG). To obtain cell aggregated pellets, 2 × 10^5^ cells (P3) were centrifuged in 96 deep well polypropylene plate (Evergreen Scientific, Vernon, CA, USA) and cultured in chondrogenic medium: high glucose-DMEM, 1 mM pyruvate (Gibco), 1% ITS +Premix (Corning), 50 μg/mL ascorbic acid (Sigma-Aldrich), 40 μg/mL L-proline (Wako, Osaka, Japan), 100 nM dexamethasone (Sigma-Aldrich), 1% antibiotic-antimitotic (Sigma-Aldrich). Experimental groups were divided by supplement with vehicle (0.1 μL/mL DMSO, serving as vehicle control), various concentrations of TD198946 (1 to 1000 nM), and/or 50 ng/mL BMP-2 (Medtronic, Dublin, Ireland). The pellets were maintained with 0.5 mL medium at 37 °C with humidified 5% CO_2_. The medium was replaced twice per week. Pellet sizes were measured from microscopic images in the same manner we reported, using NIS Elements (Nikon Instech, Tokyo, Japan) and size were described as area (mm^2^) [1, 2] (*n* = 5). For evaluation of gene expression level, 3-D pellets were cultured with chondrogenic medium for 7 days with or without 10 nM TD198946.

### 3-D scaffold culture for engineered cartilage

In Supplemental Table 1, all different combination of medium of the present study is shown. 2.5 × 10^6^ articular chondrocytes (P3) in 100 μL of culture medium (High glucose Dulbecco’s Modified Eagle Medium; DMEM, 10% fetal bovine serum and 1% antibiotic/antimycotic solution) were seeded in type I collagen scaffolds (15 mm in diameter and 3 mm in thickness, KURABO INDUSTRIES LTD., Japan) and cultured in chondrogenic medium containing with TD198946 (10 nM) and/or BMP-2 (50 or 100 ng/mL). Scaffolds were maintained with 2 mL medium at 37 °C with humidified 5% CO_2_ for two or four weeks. The medium was replaced three times per week.

### Biochemical analysis - sulfated glycosaminoglycan (sGAG) measurement

To quantitatively measure sulfated glycosaminoglycan (sGAG), the pellets (*n* = 5) were initially digested with 0.05% papain (Sigma-Aldrich) for 3 h at 65 °C with shaking and the sGAG content was measured using dimethylmethylene blue dye binding assay (Blyscan™ Glycosaminoglycan Assay Kit, Biocolor, Westbury, NY, USA) with chondroitin sulfate as a standard (kit contained). The cellularity was measured based on the double strand DNA (dsDNA) content using Qubit® 3.0 Fluorometer (Thermo Fisher Scientific, Waltham, MA, USA) and Qubit® dsDNA HS Assay kit (Thermo Fisher Scientific).

### RNA and qRT-PCR

Samples (*n* = 5) were homogenized with zirconia beads and TissueLyser (QIAGEN, Hilden, Germany) in NucleoZol (MACHEREY-NAGEL, Düren, Germany), then total RNA was extracted according to their manufacture’s protocol. RNA clean & concentrator kit (Zymo Research, Irvine, CA, USA) was used for further purification of total RNA and removal of genomic DNA. Total RNA was reverse transcribed to complementally DNA using ReverTra Ace® (Toyobo, Osaka, Japan). Quantitative RT-PCR was performed using Applied Biosystems™ Taqman® Gene Expression Assays (Thermo Fisher Scientific), Taqman® Fast Advanced Master Mix (Thermo Fisher Scientific) and StepOnePlus™ (Thermo Fisher Scientific). Target transcriptional levels were normalized to the level of glyceraldehyde 3-phosphate dehydrogenase (GAPDH) expression. The results were calculated using the relative quantitation standard curve method. The expression levels of each target genes were represented by the calculated value (2^−ΔΔct^). The information of primer is listed in Table S2.

### Implantation

For implantation, the engineered cartilage was developed in four different chondrogenic medium. Specifically, supplementing with or without 10 nM TD198946 and 100 ng/mL BMP-2 for two weeks. Twelve-week-old male athymic nude mice (BALB/cAJcl-nu/nu; CLEA japan) were anesthetized by intraperitoneal injection of a mixture of 0.3 mg/kg of medetomidine, 4.0 mg/kg of midazolam and 5.0 mg/kg of butorphanol. Total number of nude mice was 10 for the study. Bilateral subcutaneous pockets were made in the back and engineered cartilage were implanted. Origin of these cells were mini pig #2. We made four different groups (*n* = 5); vehicle alone, vehicle + BMP-2, TD198946 alone, TD198946 + BMP-2. Total number of implanted engineered cartilage was 20. At four weeks after surgery, the mice were sacrificed and the implanted engineered cartilage was used for histological analysis.

### Histological evaluation and immunohistochemistry

The samples were fixed in 4% paraformaldehyde, followed by dehydrating with ethanol, clearing with xylene, embedding in paraffin wax and 5 μm sections were prepared. The sections were stained with safranin-O/fast green/iron hematoxylin staining for sulfated GAGs. Immunohistochemical (IHC) analyses for type I collagen, type II collagen, and type X collagen were performed on sections using primary antibodies (type I collagen; Abcam plc, UK, type II collagen; Kyowa Pharma Chemical, Japan, type X collagen; Abcam plc, UK) [1]. The deparaffinized sections were incubated in 3% H_2_O_2_ for 10 min to block endogenous peroxidase activity, followed by incubation in proteinase K (DAKO, Glostrup, Denmark) for 5 min for antigen retrieval, and then in 10% goat serum for 30 min to avoid non-specific binding of primary antibodies. Primary antibody was applied to each section and incubated for 1 h. Detection was then performed using Histofine® Simple Stain™ MAX PO (MULTI; Nichirei Bioscience, Tokyo, Japan) and Simple Stain™ DAB Solution (Nichirei Bioscience). All procedure was done at room temperature, and during each step, the sections were washed three times with 0.1% Tween 20 in PBS for 5 min. Immunostaining for type I collagen, type II collagen, and type X collagen were assessed with Aperio CS2 (Leica Microsystems, Wetzlar, Germany) based on positive stained area.

### Statistics

Student T-test or two-way ANOVA test or Tukey-Kramer test were performed for comparison of 2 groups or multiple groups. All statistical analysis was performed using BellCurve for Excel® (Social Survey Research Information Co., Ltd., Tokyo, Japan). All data are presented as mean ± standard deviation. For analyses, *p* < 0.05 was used to indicate statistical significance.

## Results

### TD198946 upregulated chondrogenic gene expression and enhanced redifferentiation of dedifferentiated chondrocytes in pellet culture

**Fig. 1:**
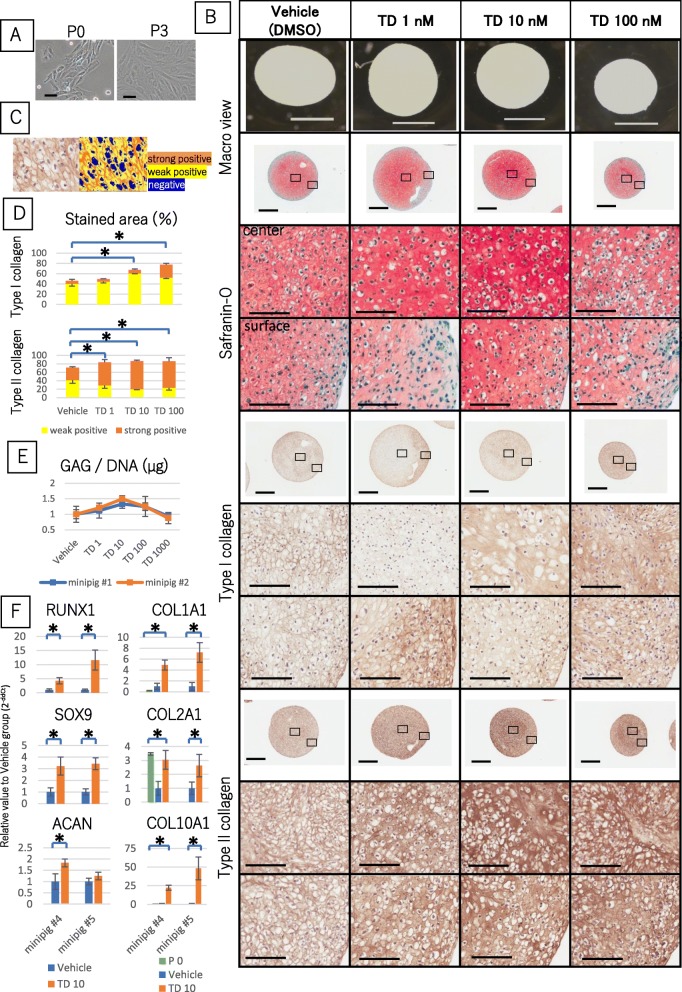
The effects of TD198946 on chondrogenic pellet culture. **a** Microscopic view of expanded chondrocytes at P0 and P3. Scale bars = 100 μm. **b** Macroscopic views of pellets treated with vehicle, 1 nM to 100 nM TD198946 at day 14. Scale bars = 1 mm. Histological images for Safranin-O, IHC for type I collagen II collagen at day 14. Scale bars = 500 μm (Low magnification: upper panels) and 100 μm (High magnification: below Low magnification). Upper images: high magnification views of central zone of pellet. Lower images: high magnification views of surface zone of pellet. High magnification panels are magnified images of the specific zone represented by the square on the low magnification view. **c**, **d** Quantitative area showing measurement of type I and II collagen. (*n* = 3) Mean ± SD, *: *p* < 0.05. **e**: sGAG content of pellets treated with vehicle, 1 nM to 1000 nM TD-198946 at day 14. (*n* = 5) Mean + SD. **f** Gene expression analysis of pellets at day 7 (*n* = 5) and chondrocyte at P0 (*n* = 5). Mean ± SD, *: *p* < 0.05. *Abbreviations: IHC, Immunohistochemistry*

Microscopically, chondrocytes at day 7 after first expanding (P0) exhibited a rounded shape, on the other hand, chondrocyte at day 28 after first expanding (P3) exhibited a spindle shape (Fig. 1a). Histological analysis of the vehicle group did stain slightly for type I and II collagen though pellets supplemented with 10 nM TD198946 stained strongly with Safranin-O and chondrocytic lacuna were particularly observed. We noted Type I collagen to be upregulated for pellets supplemented with concentrations above 100 nM of TD198946. The total stained area of IHC of type I collagen in 10 and 100 nM TD198946 groups were significantly higher than that of the vehicle group and the 100 nM TD198946 pellets stained stronger than the 10 nM treated pellets. Strong staining of type II collagen was noted over 1 nM TD198946. Type II collagen staining was shown to increase in 1 to 100 nM treated pellet than vehicle. Although, no significant difference was noted between that three groups (Fig. 1b, c, d) (*n* = 3). The relative values of GAG/DNA in each group against the vehicle group is shown in Fig. 1e (*n* = 5). There were no significant differences in GAG/DNA quantity though the highest content was noted in 10 nM TD198946 added pellets. Based on the results that 100 nM TD198946 tended to increase type I collagen, strong staining of type II collagen was noted over 1 nM TD198946 and highest content of GAG/DNA was noted in 10 nM TD198946, it was therefore decided to use the concentration of 10 nM TD198946 for further experimentation.

By adding TD198946, gene expression levels of RUNX1, SOX9, COL1A1, COL2A1 and COL10A1 were significantly higher than the vehicle group in both mini pig #4 and #5. There was however significant difference in ACAN expression only in mini pig #4. The result of ACAN was not replicable. Compared to the P0 group, gene expression levels of COL1A and COL10A1 were significantly higher on addition of TD198946. Gene expression level of COL2A1, which was lowered in vehicle group, got close to that of P0 group by adding TD198946. Each relative value to vehicle group were shown in Fig. 1f (*n* = 5). Up-regulation of COL2A1 and COL10A1 suggest redifferentiaion of dedifferentiated chondrocyte. Thus, it was noted that TD198946 enhanced the redifferentiation of dedifferentiated chondrocytes in chondrogenic pellet culture.

### BMP-2 enhanced effect of TD198946 for chondrogenic differentiation

**Fig. 2:**
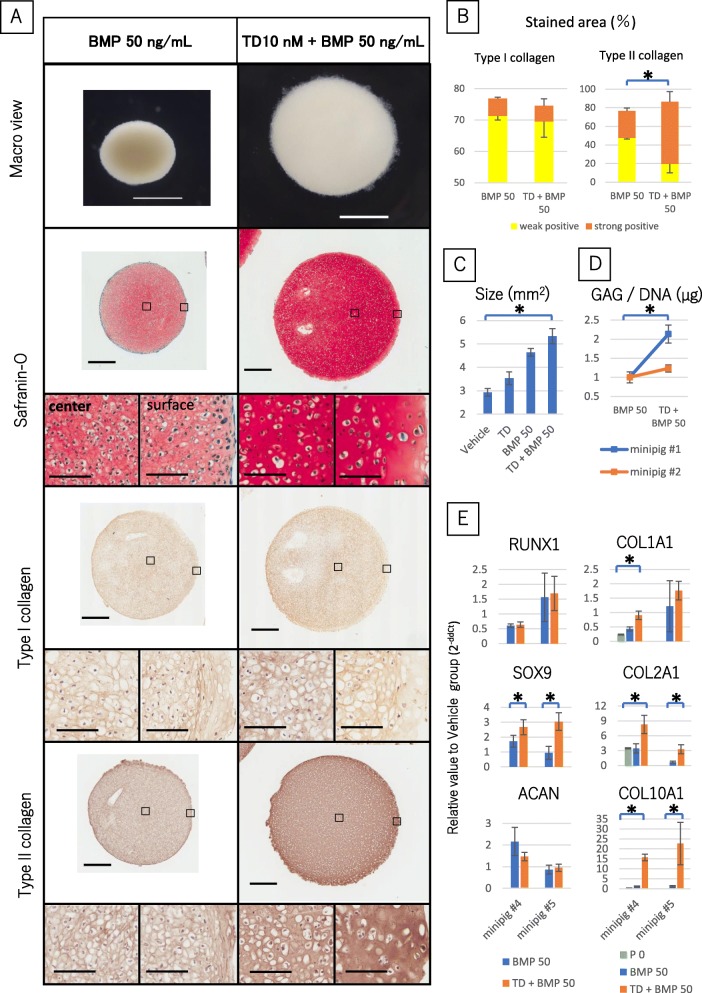
The effects of combination of TD198946 and BMP-2 on chondrogenic pellet culture. **a** Macroscopic views of pellets treated with 50 ng/mL BMP-2 alone (Left panels) and combination of 10 nM TD198946 and 50 ng/mL BMP-2 (Right panels) at day 14. Scale bars = 1 mm. Histological images for Safranin-O, IHC for type I and II collagen at day 14. Scale bars = 500 μm (Low magnification: upper panels) and 100 μm (High magnification: left and right panels). Left: high magnification view of central zone of pellet. Right: high magnification view of surface zone of pellet. High magnification panels are magnified images of the specific zone represented by the square on the low magnification view. **b** Quantitative area measurement of type I collagen and type II collagen. (*n* = 3) Mean ± SD, *: *p* < 0.05. **c** Measurement of pellet size (mm2). (*n* = 5). Mean ± SD, *: *p* < 0.05 (D): sGAG content of cultured pellets at day 14. (Vehicle + 50 ng/mL BMP-2, 10 nM TD + 50 ng/mL BMP-2) (*n* = 5). Mean ± SD, *: *p* < 0.05 **e** Gene expression analysis of cultured pellets at day 7 (*n* = 5) and chondrocyte at P0 (*n* = 5). Mean ± SD, *: *p* < 0.05. Abbreviations: IHC, Immunohistochemistry

In the following studies, concentration of added TD198946 was fixed at 10 nM. It was noted that pellets treated with 10 nM of TD198946 were significantly larger than those in the vehicle group and that pellets combined with TD198946 and 50 ng/mL BMP-2 were significantly larger than those cultured with 50 ng/mL BMP-2 alone (*n* = 5). Pellets culture with both TD198946 and 50 ng/mL BMP-2 also exhibited stronger Safranin-O staining compared with those stimulated by TD198946 or 50 ng/mL BMP-2 alone. Chondrocyte lacunas were noted in groups with 50 ng/mL BMP-2 alone and in combination with TD198946. There was a minimal expression of Type I collagen on IHC in the 50 ng/mL BMP-2 alone and combination group. There was however a strong expression of type II collagen in the TD198946 alone and combination group which was stronger than the 50 ng/mL BMP-2 alone group. Type I collagen staining was similar in the 50 ng/mL BMP-2 alone and combination group but that of type II collagen was significantly higher in the combination group (*n* = 3) (Fig. 2a-c). The GAG content (relative value against 50 ng/mL BMP-2 alone group) was significantly increased by the combination of TD198946 and 50 ng/mL BMP-2 (Fig. 2d) (*n* = 5). Gene expression (relative value against for 50 ng/mL BMP-2 alone group) of SOX9, COL2A1, and COL10A1 in the combination group was higher than that of the 50 ng/mL BMP-2 alone group. Pellets stimulated with the combination of TD198946 and 50 ng/mL BMP-2 exhibited a more hyaline cartilage-like morphology. The significant increase in the expression of RUNX1 genes demonstrated by TD198946 was not noted in the presence of 50 ng/mL BMP-2. The result of COL1A1 was not replicable. The significant increase in the expression of RUNX1 and COL1A1 genes demonstrated by TD198946 was not noted in the presence of 50 ng/mL BMP-2. There was no significant difference in ACAN expression as well as Fig. 1f. Gene expression level of COL2A and COL10A1 were significantly higher than P0 group by adding TD198946. Each relative value to 50 ng/mL BMP-2 alone group is shown in Fig. 2e (*n* = 5).

### TD198946 induced hyaline cartilage like tissue in 3-D collagen scaffold culture

**Fig. 3:**
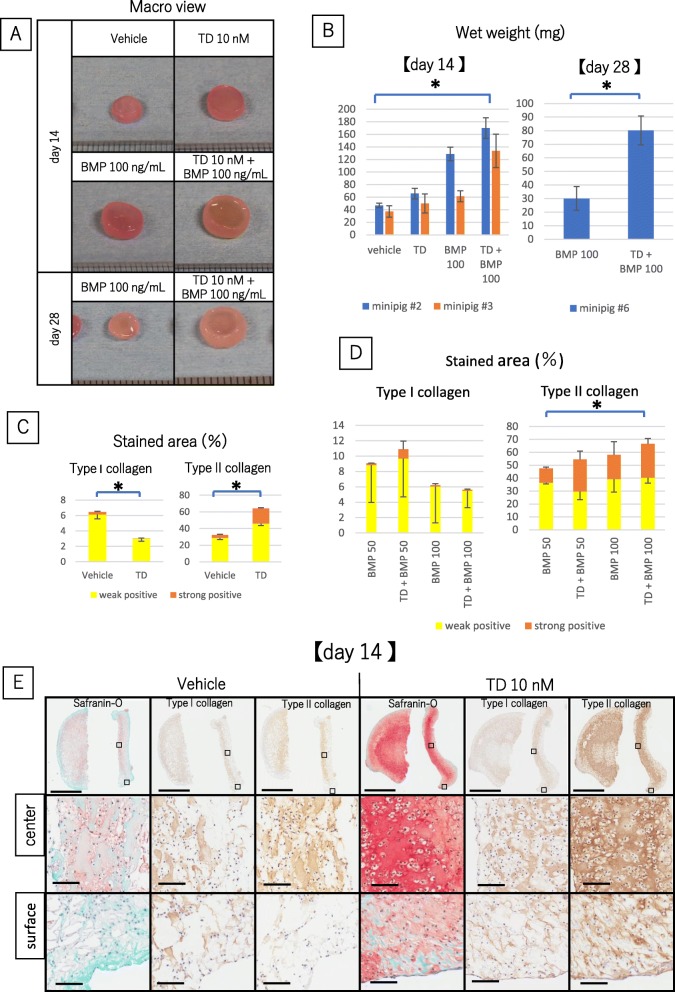
The effects of TD198946 and combination of TD198946 and BMP-2on engineered cartilage generation. **a** Macroscopic views of engineered cartilages treated with vehicle or 10 nM TD198946 at day 14. Engineered cartilages treated with 100 ng/mL BMP-2 alone and combination of 10 nM TD198946 and 100 ng/mL BMP-2 at day 14 and 28. **b** Wet weight of engineered cartilages treated with vehicle, 10 nM TD198946, 100 ng/mL BMP-2 alone and combination of 10 nM TD198946 and 100 ng/mL BMP-2 at day 14. (*n* = 5). Mean ± SD, *: *p* < 0.05. The wet weights of engineered cartilages treated with 100 ng/mL BMP-2 alone and combination of 10 nM TD198946 and 100 ng/mL BMP-2 at day 28. (*n* = 6). **c** Quantitative area measurement of type I and II collagen. (*n* = 3) Mean ± SD, *: *p* < 0.05. **d** Quantitative area measurement of type I collagen and type II collagen. (*n* = 3) Mean ± SD, *: *p* < 0.05 **e** Histological images for Safranin-O, IHC for type I and II collagen (Low and high magnification views) of engineered cartilage at day 14. Left side panels: treated with vehicle. Right side panels: treated with 10 nM TD198946. Scale bars = 2 mm (Low magnification: upper panels) and 100 μm (High magnification: below Low magnification). Upper images: high magnification view of central zone. Lower images: high magnification view of surface zone of engineered cartilage. High magnification panels are magnified images of the specific zone represented by the square on the low magnification view

During 3-D collagen scaffold culture it was noted that the wet weight of the engineered cartilage cultured with TD198946 was significantly higher than those of the vehicle group (Fig. 3a, b) (*n* = 5). In TD198946 group, Safranin-O staining stained strongly in the central zone, while less staining was noted in the vehicle group. Cultures with 10 nM TD198946 stained strongly for type II collagen compared to the vehicle group and all cultures with TD198946 demonstrated chondrocytic lacunae. IHC for type I collagen showed less staining in the TD198946 added groups when compared to the vehicle group (Fig. 3e). These finding were supported by the stained area (Fig. 3c) (*n* = 3). Compared with the vehicle group the engineered cartilage in the 10 nM TD198946 group showed histological characteristics of hyaline cartilaginous tissue.

### The combination of TD198946 and BMP-2, enhanced chondrogenic differentiation of engineered tissue from cell seeded scaffold

**Fig. 4:**
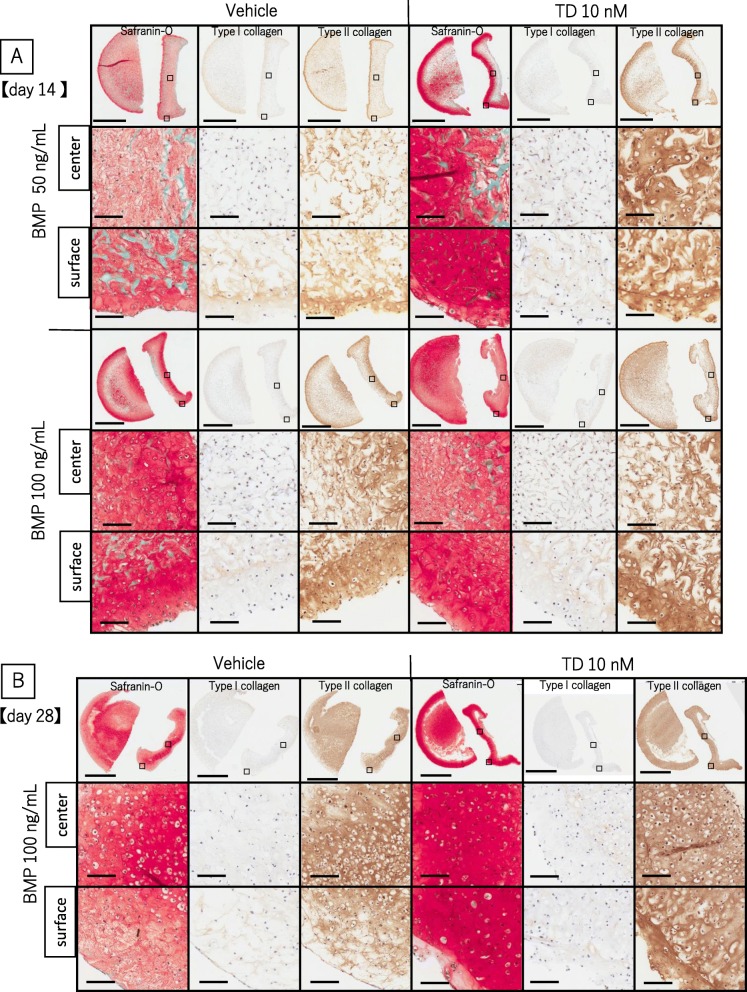
The effects of combination of TD198946 and BMP-2 on engineered cartilage generation. **a** Histological images for Safranin-O, IHC for type I and II collagen (Low and high magnification views) of engineered cartilage at day 14. Upper three row panels: treated with 50 ng/mL BMP-2. Lower panels: treated with 100 ng/mL BMP-2. Left panels: treated with vehicle. Right panels: treated with 10 nM TD198946. Scale bars = 2 mm (Low magnification: upper panels) and 100 μm (High magnification: below Low magnification). Upper high magnification views are of central zone and lower high magnification views are of surface zone of engineered cartilage. High magnification panels are magnified images of the specific zone represented by the square on the low magnification view. **b** Histological images for Safranin-O, IHC for type I and II collagen of engineered cartilage at day 28. Scale bar and panels are displayed in the same manner as **a**. *Abbreviations: IHC, Immunohistochemistry*

We noted combining TD198946 and BMP-2 enhanced chondrogenic differentiation of the generated tissue in the cell-seeded scaffold as well as pellet culture. BMP-2 at concentrations of 50 or 100 ng/mL were combined with TD198946. The wet weight of the engineered cartilage treated with a combination of TD198946 and 100 ng/mL BMP-2 was significantly higher than the 100 ng/mL BMP-2 alone group at day 14 and day 28. Both groups were significantly higher than vehicle and 10 nM TD198946 group at day 14 (Fig. 3a, b) (*n* = 5). Safranin-O staining stained homogeneously in the combination group of TD198946 and 100 ng/mL BMP-2, while the stain was limited in the outer zone in other study groups. IHC for type I collagen was almost negative or weak in all groups but type II collagen was homogeneously detected in the combination of TD198946 and 100 ng/mL BMP-2 groups (Fig. 4a). Regarding stained area no significant difference was noted in type I collagen, but the stained area of type II collagen in the combination of TD198946 and 100 ng/mL BMP-2 group was the highest of the four groups (Fig. 3d) (*n* = 3). We, therefore, noted that the combination of TD198946 and 100 ng/mL BMP-2 had a positive synergistic effect in enhancing chondrogenic differentiation of engineered cartilage in our study and revealed superior results when compared to our other study groups. These effects were also noted in a long-term culture up to day 28. The wet weight of engineered cartilage in the combination group was significantly higher than that of BMP-2 alone group at day28 (Fig. 3a, b) (*n* = 6). Strong staining for type II collagen was displayed in both 100 ng/mL BMP-2 alone and the combination group, though the combination group stained stronger than the BMP-2 alone group (Fig. 4b).

### Engineered cartilage treated with combination of TD198946 and BMP-2 displayed partial thinning and cartilage ossification but maintained ECM post-implantation

**Fig. 5:**
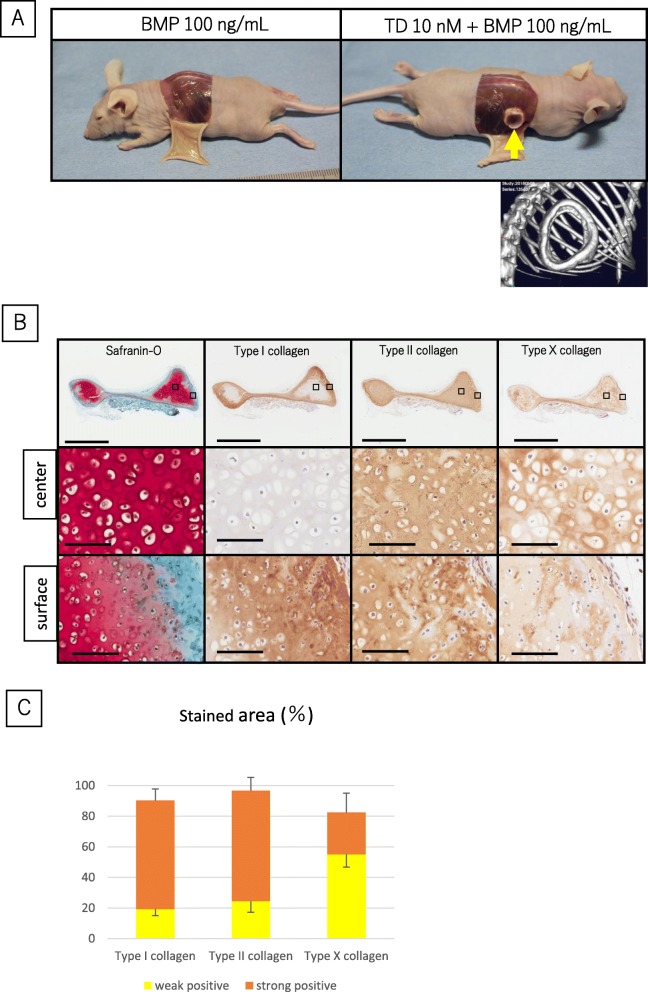
An evaluation of engineered cartilage treated with TD198946 and BMP-2 in vivo. **a** Macroscopic views of sacrificed mice and implanted engineered cartilages. (*n* = 5) 3-D μ-CT scan image of implanted engineered cartilages treated with combination of 10 nM TD198946 and 100 ng/mL BMP-2 below the macro view. (*n* = 2) **b** Histological images for Safranin-O, IHC for type I, II and X collagen. Scale bars = 2 mm (Low magnification: upper panels) and 100 μm (High magnification: below Low magnification). Upper images: high magnification views of central zone. Lower images: high magnification views of surface zone. High magnification panels are magnified images of the specific zone represented by the square on the low magnification view. **c** Quantitative area measurement of type I, II and X collagen. (*n* = 5) Mean ± SD, *: *p* < 0.05. *Abbreviations:* μ-CT, micro-computed tomography*; IHC, Immunohistochemistry*

On implantation, the groups added with 100 ng/mL BMP-2 alone were mostly absorbed and disappeared on the at the end of 4 weeks. For that reason, we could not evaluate histologically BMP-2 alone group. However, engineered cartilage treated with a combination of TD198946 and 100 ng/mL BMP-2 did not disappear and remained at the implantation site. There was however a notable thinning and translucency in the central zone (Fig. 5a) (*n* = 5). μCT scan (3-D-micro X-ray CT; IKEDA SCIENTIFIC Co. Ltd., Japan) confirmed features of cartilage ossification in the surface zone (mini pig #2; *n* = 2/2), which may be the reason the tissue turned an ivory white colour. On histological evaluation, Safranin-O stained strongly in the central zone though reduced staining was noted in the surface zone. Under high magnification, hypertrophic chondrocytes were noted in the central and surface zone. The surface zone stained homogeneously for type I and II collagen and heterogeneously for type X collagen (Fig. 5b). The stained area of type I and II collagen is shown in Fig. 5c (*n* = 5) and was increased after ectopic implantation compared to that of engineered tissue pre-implantation shown in Fig. 3d. Although partial cartilage ossification was exhibited, the engineered cartilage treated with the combination of TD198946 and BMP-2 in vitro exhibited ECM which have characteristics of hyaline cartilage-like tissue such as GAG and type II collagen in central zone.

## Discussion

In the present study, we evaluated whether the addition of TD198946 to a three-dimensional culture or engineered cartilage would improve dedifferentiated chondrocyte differentiation. Our study demonstrated that supplementation with TD198946 is a feasible method to enhance chondrogenic differentiation in pellet cultures and chondrocyte-based tissue engineering. TD198946 exhibited a synergistic effect when combined with BMP-2 resulting in the implanted engineered cartilage being less absorbed and having better quality histology resembling that of hyaline cartilage.

TD198946 facilitated the redifferentiation of dedifferentiated chondrocytes and generated hyaline cartilaginous tissue by deposition of glycosaminoglycan and type II collagen in the pellet culture and engineered cartilage. Though the exact mechanism of action by which TD198946 is functions is unclear, it was previously demonstrated that TD198946 upregulated Runx1, Sox9 and Col2a1 in C3H10T1/2 cells, ATDC5 cells and mice chondrocytes [19]. Our study was in accordance with these results, but we also noticed the upregulation of COL1A1 and COL10A1. This indicated that the addition of TD198946 led the mini pig chondrocytes used in our study to later stages of chondrogenesis followed by hypertrophic change and cartilage ossification, unlike the report by Yano et al. [19]. The variation in results was thought to be due to the difference in cell source and culture environment being a 3-D culture as opposed to monolayer culture. In the present study, ACAN was not upregulated by adding TD198946 and combination of 10 nM TD198946 and 50 ng/mL BMP-2, while GAG synthesis was facilitated by TD198946 alone and combination. The reason for this is unclear and further evaluation is required in the planned future study.

The type I collagen stained area was increased in the TD198946 pellet culture but decreased in the engineered cartilage. This suggests that cell seeding on a scaffold was a favorable environment for inducing hyaline like tissue when compared to pellet culture. Though we were able to produce hyaline like cartilage tissue in the engineered cartilage using TD198946, on subcutaneous implantation the engineered tissue was absorbed (Data not showed), indicating the level of chondrogenic differentiation was unsatisfactory.

On combining TD198946 and BMP-2 we noticed the increased deposition of glycosaminoglycan and type II collagen in the pellet culture. In the engineered cartilage this resulted in the production of a homogeneous ECM. There are reports of engineered cartilage developed from dedifferentiated chondrocyte with the addition of TGF-β over four weeks [12, 17]. In the present study, we were able to generate comparable cartilaginous tissue in a shorter period of two weeks of chondrogenic culture, in pellet and seeded scaffold cultures in the presence of TD198946 and BMP2. The combination of these two molecules facilitated a more efficient chondrogenic differentiation of the engineered cartilage and maintained the tissue ECM even after implantation. On the other hand, cultures treated with BMP-2 alone were mostly absorbed.

Though combining TD198946 and BMP-2 resulted in a better homogeneous chondrogenic differentiation in vitro, on implantation thinning of the central zone and ossification of the central zone was noted. We performed a preliminary experiment in a mini pig model, adding both TD198946 and BMP-2 to engineered cartilage to treat an osteochondral knee joint lesion (Data not shown). During implantation, we used a biopsy punch to adjust the engineered cartilage size to that of the knee defect size. This resulted in the engineered cartilage to divide into two layers. The cause of this was thought to be due to the heterogeneous distribution of the seeded chondrocytes in the collagen sponge scaffold and lack of ECM in the central zone of the engineered cartilage. The various scaffold materials and 3-D culture methods currently available should be evaluated for their ability to induce homogeneous chondrogenic differentiation and ECM production in engineered cartilage.

Concerning growth factors, many types have been under study such as TGF-β and IGF-1 to determine their ability for chondrogenic differentiation or redifferentiation of dedifferentiated chondrocyte [5, 7, 11]. Combining growth factors such as BMP-2 and TGF-β3 with TD198946 has also proven to be advantageous for chondrocyte culture as demonstrated in our study and another by Chijimatsu et al. [2]. We can, therefore, ascertain that such growth factors are feasible for chondrogenic differentiation, though TGF-β and BMP are known to upregulate type 1 and type X collagen as well as type II collagen. This is the reason for the regenerate tissues to progress toward hypertrophic and subsequent cartilage ossification [5, 6]. This may be the same reason for the cartilage ossification noted in our implanted engineered cartilage.

We have reported that TD198946 upregulated RUNX1 which is a regulator of Sox9 the main gene expressed for chondrogenic differentiation in a 2-D assay using C3H10T1/2 cells [19]. In the present study, RUNX1 was upregulated by TD198946 alone which upregulated expression of SOX9 and COL2A1 in pellet culture on day 7. Though there was similar upregulation of SOX9 and COL2A1 in the combination group, TD198946 did not up-regulate RUNX1 in the presence of BMP-2. The combination group exhibited stronger Safranin-O staining and IHC for type II collagen without the up-regulation of RUNX1. It has been reported that RUNX1 expression increases in the early stages of chondrogenic differentiation and then decreases with subsequent chondrocyte hypertrophy and cartilage ossification [18, 19]. Our results suggest that BMP-2 promoted chondrogenic differentiation from the early stage to later stages without the upregulation of RUNX1.

## Limitations

Multiple examinations should be evaluated with chondrocytes isolated from a single mini pig. In our study, chondrocytes were isolated from multiple mini pigs as the harvest from a single mini pig was limited and insufficient for serial examinations. Therefore, only limited samples could be prepared for statistical analysis and implantation in nude mice. The same experiments in nude mice should be done with human cells to confirm their susceptibility to the compound too. The cells used here are from mini pig. Mechanical testing was not performed on the engineered cartilage. We did not evaluate the effects of implantation of the engineered cartilage in a knee joint in vivo study.

## Conclusions

Our study demonstrated that TD198946 supplementation is a feasible method to enhance chondrogenic capacities in pellet culture and chondrocyte-based tissue engineering. We also noted TD198946 combined with BMP-2 results in superior stability and histological characteristics of the engineered cartilage after implantation.

If the same response to TD198946 will be confirmed in human cells, this approach could be a promising addition to develop better quality engineered cartilage tissue in a clinical setting.

## Supplementary information

**Additional file 1: Supplemental Table 1.** Group list.

**Additional file 2: Supplemental Table 2.** Taqman assay list.

**Additional file 3.**

## Data Availability

The data that support the findings of the current study available from the corresponding author on reasonable request.
