# Supplementary material for: Enhancement of chondrogenic differentiation supplemented by a novel small compound for chondrocyte-based tissue engineering
Source: J Exp Orthop. 2020 Mar 7;7:10. doi: 10.1186/s40634-020-00228-8 (PMC7060980; doi:10.1186/s40634-020-00228-8)
Supplement: Supplementary file 1 — Additional file 1: Supplemental Table 1. Group list. [file 40634_2020_228_MOESM1_ESM.docx]

Supplemental Table 1

Group list
