# Supplementary material for: Enhancement of chondrogenic differentiation supplemented by a novel small compound for chondrocyte-based tissue engineering
Source: J Exp Orthop. 2020 Mar 7;7:10. doi: 10.1186/s40634-020-00228-8 (PMC7060980; doi:10.1186/s40634-020-00228-8)
Supplement: Supplementary file 2 — Additional file 2: Supplemental Table 2. Taqman assay list. [file 40634_2020_228_MOESM2_ESM.docx]

Supplemental Table 2

Taqman assay list

| Gene symbol | Assay code |
| --- | --- |
| GAPDH | Ss03375629_u1 |
| COL2A1 | Ss03373344_g1 |
| ACAN | Ss03374823_m1 |
| SOX9 | Ss03392406_m1 |
| RUNX1 | Ss04322790_m1 |
| COL1A1 | Ss03373340_m1 |
| COL10A1 | Ss03391766_m1 |
