# Supplementary figures and images for: Enhancement of chondrogenic differentiation supplemented by a novel small compound for chondrocyte-based tissue engineering
Source: J Exp Orthop. 2020 Mar 7;7:10. doi: 10.1186/s40634-020-00228-8 (PMC7060980; doi:10.1186/s40634-020-00228-8)

Additional figure

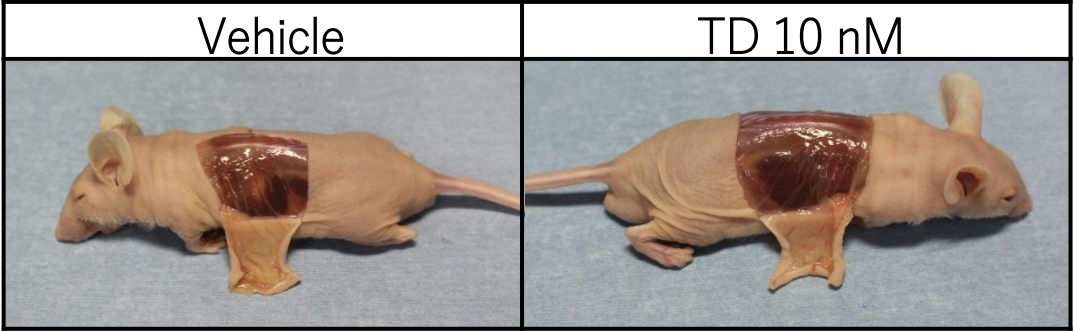

Supplement: Supplementary file 3 — Additional file 3. [file 40634_2020_228_MOESM3_ESM.pdf]
